# ﻿Chromosomes of the genus *Arge* Schrank, 1802 (Hymenoptera, Argidae): new data and review

**DOI:** 10.3897/compcytogen.17.115485

**Published:** 2023-12-21

**Authors:** Vladimir E. Gokhman

**Affiliations:** 1 Botanical Garden, Moscow State University, Moscow 119234, Russia Moscow State University Moscow Russia

**Keywords:** Chromosome morphometry, karyotypes, sawflies

## Abstract

Results of the chromosome study of 12 sawfly species of the genus *Arge* Schrank, 1802 are reviewed, including new data on the karyotypes of *A.ciliaris* (Linnaeus, 1767) and *A.enodis* (Linnaeus, 1767) with n = 10. Moreover, the same chromosome number, n = 10, is found in *A.ustulata* (Linnaeus, 1758), for which n = 8 was previously reported. In addition, n = 8 is confirmed in *A.gracilicornis* (Klug, 1814). The results of the morphometric analysis of chromosome sets of these four species are given. In the genus *Arge*, haploid chromosome numbers of n = 8, 10, 11 and 13 were found. Among these sawflies, n = 8 appeared to be the most frequent chromosome number, followed by n = 10. The known data of the chromosome study of these insects are summarized and discussed in the light of phylogeny and taxonomy of the genus *Arge*.

## ﻿Introduction

*Arge* Schrank, 1802 is the most speciose genus of the family Argidae, which is, in turn, the second largest group of its kind among sawflies (Symphyta) (Taeger et al. 2018). The genus *Arge* currently includes more than 400 described species, with about 180 members of the genus occurring in the Palaearctic (Taeger et al. 2018). To date, chromosomal data for Argidae are known only for ten *Arge* species (Naito 1982; Westendorff 2006). For most of them, certain additional information on the karyotype structure is also available. In the present paper, I have recently examined karyotypes of several members of this genus, including two newly studied species. In another two species, either the existing chromosome number was confirmed, or, unexpectedly, a different n value was found. Since some members of the genus *Arge* appeared to have superficially similar karyotypes, morphometric analysis of the chromosome sets, which could find some hidden interspecific differences, was also undertaken. The existing results of the chromosome study of the genus *Arge* are summarized and discussed in the light of phylogeny and taxonomy of these sawflies (see below).

## ﻿Material and methods

Adult female sawflies of the genus *Arge* were collected by the author in the wild, mostly on the flowers of umbelliferous plants (Apiaceae) in Ozhigovo, Moscow, Russia (55°28'N, 36°52'E) in 2022–2023 (Table 1). The sawflies were initially identified by the author, the identifications were then checked by Sergey A. Basov (Zoological Institute, Russian Academy of Sciences, St. Petersburg, Russia). Voucher specimens are deposited in the collection of the Zoological Museum of Moscow State University (Moscow, Russia).

**Table 1.** Relative lengths (RLs) and centromeric indices (CIs) of chromosomes of four *Arge* species (mean ± SD).

Chromosome no.	*A.gracilicornis*		*A.enodis*		*A.ciliaris*		*A.ustulata*	
	RL	CI	RL	CI	RL	CI	RL	CI
1	27.07 ± 1.32	41.80 ± 3.88	15.04 ± 0.87	40.68 ± 3.37	23.22 ± 0.92	42.08 ± 4.00	25.98 ± 1.34	43.66 ± 4.35
2	13.59 ± 0.54	44.52 ± 4.10	12.68 ± 0.88	41.10 ± 4.45	13.23 ± 2.13	41.45 ± 4.31	14.49 ± 0.82	40.81 ± 4.23
3	12.31 ± 0.60	46.94 ± 1.76	11.39 ± 0.58	41.32 ± 4.80	10.41 ± 0.34	42.60 ± 3.77	11.18 ± 0.41	43.49 ± 3.31
4	11.67 ± 0.46	45.37 ± 4.19	10.66 ± 0.35	40.03 ± 4.48	9.25 ± 0.54	34.34 ± 3.35	10.52 ± 0.47	45.44 ± 3.72
5	10.38 ± 0.64	42.80 ± 3.12	9.56 ± 0.47	37.81 ± 4.78	8.48 ± 0.45	38.79 ± 2.98	9.14 ± 0.36	42.41 ± 4.13
6	9.82 ± 0.69	42.64 ± 3.94	9.22 ± 0.47	37.46 ± 4.63	8.28 ± 0.51	37.20 ± 4.64	7.12 ± 0.57	41.96 ± 4.93
7	9.08 ± 0.48	46.40 ± 2.75	8.67 ± 0.47	41.74 ± 3.48	7.57 ± 0.49	38.67 ± 3.96	6.03 ± 0.27	43.26 ± 4.69
8	6.08 ± 0.33	0	8.06 ± 0.36	38.74 ± 4.05	7.09 ± 0.50	36.38 ± 4.83	5.48 ± 0.33	42.58 ± 3.96
9	–	–	7.56 ± 0.31	44.31 ± 3.40	6.49 ± 0.54	41.01 ± 3.70	5.25 ± 0.35	43.86 ± 3.70
10	–	–	7.16 ± 0.37	39.28 ± 4.79	5.98 ± 0.42	43.79 ± 3.09	4.81 ± 0.40	44.45 ± 4.91

Chromosomal preparations were obtained from embryos forming inside the developing eggs, generally following the protocols used by Naito (1982) and Imai et al. (1988) with a few modifications. Specifically, mature eggs were extracted from adult females and put inside small Petri dishes on a filter paper soaked with distilled water. These eggs were kept for about three days at room temperature. During that time, sawfly embryos developed inside these eggs. These embryos were first dissected in 0.5% hypotonic sodium citrate solution containing 0.005% colchicine, and then transferred to a fresh portion of hypotonic solution and incubated for 30 min at room temperature. After that, the material was transferred onto a pre-cleaned microscope slide using a Pasteur pipette and then gently flushed with Fixative I (glacial acetic acid: absolute ethanol: distilled water 3:3:4). The tissues were disrupted using dissecting needles in an additional drop of Fixative I. Another drop of Fixative II (glacial acetic acid: absolute ethanol 1:1) was applied to the center of the area, and the more aqueous phase was blotted off the edges of the slide. The same procedure was then performed with Fixative III (glacial acetic acid). The slides were dried for approximately half an hour and stored at room temperature. The preparations were stained overnight with a freshly prepared 3% Giemsa solution.

Haploid mitotic divisions were studied and photographed using an optic microscope Zeiss Axioskop 40 FL fitted with a digital camera Axiocam 208 color (Carl Zeiss, Germany). To produce illustrations, the resulting images were handled with image processing programs ZEN version 3.0 (blue edition) and GIMP version 2.10. Chromosomes were measured on ten metaphase plates of all studied species using KaryoType software version 2.0 and then classified according to the guidelines provided by Levan et al. (1964), i.e., as metacentrics (M), submetacentrics (SM), subtelocentrics (ST) and acrocentrics (A).

## ﻿Results

**Figure 1.**
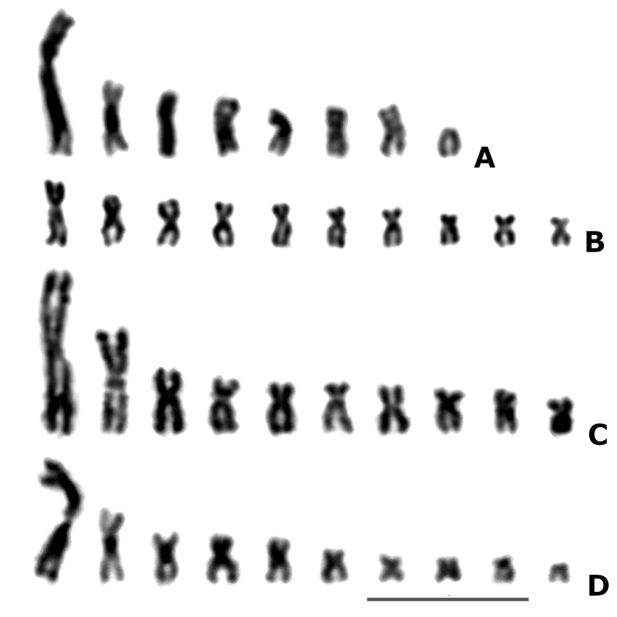
Haploid karyograms of *Arge* species **A***A.gracilicornis***B***A.enodis***C***A.ciliaris***D***A.ustulata*. Scale bar: 10 μm.

*Argegracilicornis* (Klug, 1814) (n = 8). Seventeen embryos obtained from four females were examined. Most chromosomes are metacentric/submetacentric, but the shortest one is an acrocentric (Fig. 1A, Table 2). The first chromosome is very large, about twice as long as the second one, which is, in turn, more than twice as long as the last acrocentric (Table 1). All chromosomes, except the largest and the smallest, form a continuous gradation in length.

**Table 2.** Karyotypes of *Arge* species.

Species	n(2n)	Chromosomal formula, n	Region	Reference
*A.ciliaris* (Linnaeus, 1767)	10	3M + 7M/SM	European Russia	Present paper
*A.clavicornis* (Fabricius, 1781)	8	8M^†^	Eastern Canada	Maxwell 1955, 1958
*A.cyanocrocea* (Förster, 1771)	11	6M + 5SM	Eastern Germany	Westendorff and Taeger 2002
*A.enodis* (Linnaeus, 1767)	10	2M + 8M/SM	European Russia	Present paper
*A.gracilicornis* (Klug, 1814)	8	7M + 1ST	Eastern Germany	Westendorff and Taeger 2002
	8	7M + 1A	European Russia	Present paper
*A.jonasi* (Kirby, 1882)	10	5M + 5M/SM^†^	Japan	Naito 1976
*A.melanochra* (Gmelin, 1790)	10	5M + 5SM	Eastern Germany	Westendorff and Taeger 2002
*A.nigripes* (Retzius, 1783)	13	4M + 4M/SM + 5ST/A^†^	Eastern Germany	Westendorff and Taeger 2002
*A.nigronodosa* (Motschulsky, 1860)	8	4M + 3M/SM + 1A^†^	Japan	Naito 1982
*A.pagana* (Panzer, 1798)	(16)	7M + 1M/SM	Eastern Germany	Westendorff and Taeger 2002
*A.pectoralis* (Leach, 1817)	8	8M^†‡^	Eastern Canada	Maxwell 1955, 1958
*A.ustulata* (Linnaeus, 1758)	8(16)	?	Scotland, UK	Greenshields 1937
	10	8M + 2M/SM	European Russia	Present paper

^†^ Extrapolated from images given in the references. ^‡^ Although both images given by Maxwell (1955) for this species show n = 7, they strongly differ in the relative size of the largest chromosome. However, this author defines both *A.clavicornis* and *A.pectoralis* as “cytologically homogeneous” (Maxwell 1955, 1958), and I therefore follow Westendorff (2006) who suggested n = 8 for the latter species.

*A.enodis* (Linnaeus, 1767) (n = 10). Two embryos obtained from a single female were examined. All chromosomes are obviously biarmed, either metacentric or submetacentric (Fig. 1B, Table 2). However, unlike the karyotype of the previous species, length of the first chromosome only slightly exceeds that of the second one (Table 1). The remaining chromosomes gradually decrease in size.

*A.ciliaris* (Linnaeus, 1767) (n = 10). Ten embryos, also obtained from a single female, were examined. As in the previous species, all chromosomes are clearly biarmed, either metacentric or submetacentric (Fig. 1C; Table 2). Similarly to *A.gracilicornis*, the first chromosome is very large, about four times longer than the last one (Table 1). In turn, the second chromosome is approximately 1.8 times shorter than the preceding one, all other elements more or less gradually decreasing in length. On most metaphase plates, a secondary constriction can be clearly seen in the pericentromeric region of the longer arm of the second chromosome.

*A.ustulata* (Linnaeus, 1758) (n = 10). Seven embryos obtained from four females were studied. The karyotype generally resembles that of *A.ciliaris* (Fig. 1D, Table 2). As in the previous species, most chromosomes, except for the first and second ones, form a continuous gradation in size, but the fifth chromosome is visibly longer than the remaining elements (Table 1).

## ﻿Discussion

Up to now, karyotypes of 12 members of the genus *Arge* have been studied. In these sawflies, haploid chromosome numbers of n = 8, 10, 11 and 13 were found (Table 2). Among these species, n = 8 appeared to be the most frequent chromosome number, followed by n = 10. Within chromosome sets of *Arge* species, metacentrics and submetacentrics usually predominate (Table 2, Westendorff and Taeger 2002), although most members of the genus with the same n values differ by their karyotype structure. For example, n = 10 is characteristic of both *A.ciliaris* and *A.enodis*, but the chromosome set of the former species contains a very large metacentric, which is absent from the karyotype of *A.enodis*. Analogously, *A.gracilicornis* and *A.nigronodosa* both have chromosome sets with n = 8, again with a large first metacentric, but the second metacentric/submetacentric chromosome of the latter species is substantially longer than that of *A.gracilicornis* (Naito 1982; Westendorff and Taeger 2002; present study). Moreover, Westendorff and Taeger (2002) identified the last chromosome of *A.gracilicornis* as a subtelocentric, which can also be clearly seen on Fig. 1 of their paper, but a shorter arm of an analogous acrocentric chromosome of apparently the same species is not visible (present paper, Fig. 1A). However, it is unclear at the moment whether this feature represents an intraspecific chromosomal polymorphism or indicates the presence of cryptic species within the *A.gracilicornis* complex. In *A.ustulata*, a common European species, possible involvement of cryptic taxa is also supposed. Specifically, n = 8 and 2n = 16 were reported in the 1930s for this sawfly species in the United Kingdom (Greenshields, 1937), whereas material from central European Russia clearly shows n = 10 (present paper). Nevertheless, wrong identification of the British material cannot be completely ruled out as well.

Given the relatively high karyotypic diversity of the genus *Arge*, it is difficult to understand what the initial karyotype for the group might look like. Judging from the most frequent chromosome numbers, the ancestral n value could be close to 8 or 10. Both these numbers fall within range of putative initial values for the superfamily Tenthredinoidea and Argidae in particular, i.e., n = 7 to 10 (Gokhman 2023). Moreover, n = 8 is the only chromosome number found in different subfamilies of Pergidae, a sister group to Argidae (Boevé et al. 2018; Gokhman 2023). Nevertheless, n = 8 and 10 alternatively predominate in two apparent *Arge* clades (Boevé et al. 2018), but the ancestral chromosome number for this group may also be substantially higher. In addition, karyotypes of various members of the genus *Arge* contain the very large first metacentric chromosome, e.g., *A.ciliaris*, *A.gracilicornis*, *A.melanochra*, *A.pagana* and *A.ustulata* (Westendorff and Taeger 2002; present paper). However, whether this chromosome represents an ancestral character state for the genus remains an open question. Analogously, little can be said at present about the possible chromosomal rearrangements underlying the process of karyotypic change within this genus. Similarly to other sawflies and Hymenoptera in general, differences between karyotypes of related *Arge* species could be explained by chromosomal fusions/fissions, deletions/duplications of the constitutive heterochromatin, translocations and/or inversions (Gokhman 2009, 2023).

Nevertheless, I believe that karyotype analysis can be successfully used in further taxonomic and phylogenetic studies of the genus *Arge* due to its high chromosomal diversity. Our results together with published karyotypic data collectively suggest that chromosome sets of most species of this group can be easily distinguished without a detailed morphometric analysis. On the other hand, this kind of analysis can be important at least in some cases, which can be judged from an example of *A.ciliaris* and *A.ustulata* (see above). This situation is generally similar to the pattern observed in other studied sawfly families, e.g., Tenthredinidae (Westendorff 2006; Gokhman 2023).
